# Effects of a Comprehensive, Multiple Risky Behavior Prevention Program on High School Students

**DOI:** 10.1155/2016/9545847

**Published:** 2016-09-08

**Authors:** Crystal Collier, Anthony J. Onwuegbuzie, Alicia LaChapelle, Jessica Davison

**Affiliations:** ^1^Department of Educational Leadership and Counseling, Sam Houston State University, Huntsville, TX, USA; ^2^The Council on Recovery, Houston, TX, USA; ^3^Department of Social Work, Texas Southern University, Houston, TX, USA

## Abstract

The purpose of this research study was to examine the effect of a multiple risky behaviors prevention program applied comprehensively throughout an entire school system involving universal, selective, and indicated levels of students at a local private high school during a 4-year period. The noncurriculum prevention program was created based upon the key elements of effective prevention programming and the need to address the growing variety of risky behaviors that the youth face today. Results (*n* = 469 to 614) indicated that 7 out of 15 risky behaviors statistically significantly decreased throughout the 4-year study, with 6 behaviors involving alcohol and drugs. However, many of the targeted non-substance-use risky behaviors displayed inconsistent prevalence rate patterns without statistically significant changes. These findings indicate that the frequency and intensity of programming for non-substance-use behaviors should be increased to a value at least equal to that of the substance-use behaviors. Implications for schools, prevention specialists, and future program development and research are discussed.

## 1. Introduction

In the United States, the average age of the first use of alcohol and marijuana is between 15 and 17 [1, 2]. In fact, adolescent alcohol and drug use increases each year during the high school period [3]. Unfortunately, early alcohol and drug use is not the only risky behavior of concern [4]. Along with prescription drug abuse [3] and binge drinking [1], adolescents also struggle with gambling [5, 6], pornography [7, 8], self-injury [9], cyberbullying [10, 11], eating disorders [12, 13], video game addiction [14], suicide [15, 16], driving while drinking [1], and dating violence [17, 18]. Furthermore, many adolescents engage in more than one of these risky behaviors simultaneously [19, 20]. The occurrence of simultaneous multiple risky behaviors is significant and increases from freshman to senior year in high school [21].

Empirical evidence of the effectiveness of prevention programs indicates that certain risky behaviors have been prevented or reduced for up to 15 years [22]. This literature suggests that comprehensive prevention programming can target and reduce multiple risky behaviors simultaneously with long-term effects [23, 24]. Furthermore, the literature suggests that when prevention programs target the entire student body, family, school, and community—rather than a targeted subset—it produces larger and longer lasting effects on the risky behaviors [25, 26]. The Institute of Medicine (IOM) defines such broad-based programs directed toward students regardless of risk as* universal* prevention programs, whereas programs that target students at risk for problem behaviors are referred to as* selective* and programs that target students who are already engaging in risky behaviors are called* indicated* [27].

Although most prevention programs in the United States are universal, they focus only on two to three risky behaviors. However, evidence indicates that integrated, multiple-risk prevention programs can be effective across a range of health risk behaviors prevalent in adolescence [23, 25, 28]. The review of the literature leads to the question of what would the effects of a comprehensive prevention program involving all three Institute of Medicine's categories (i.e., universal, selective, and indicated) and targeting multiple risky behaviors that adolescents struggle with today be. Hence, the first author's prevention team developed the Choices program integrating these components and including key elements of previously successful prevention efforts [20, 23, 26, 27]. In this study, we examined the effectiveness of the Choices program applied to a comprehensive target audience involving universal, selective, and indicated levels of students at a local private high school.

### 1.1. Program Description

**Figure 1:**
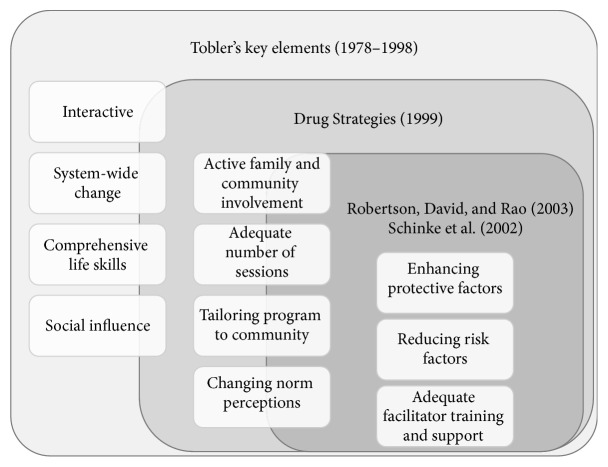
Visual representation of the key elements of prevention programs beginning with Tobler et al.'s [30] findings and including additional elements found in later research (Drug Strategies (1999) [25]; Schinke et al. (2002) [26];  Robertson et al. (2003) [31]).

The development of the Choices program was guided by empirical studies involving meta-analysis and reviews of school-based prevention models that have revealed key elements of effective prevention programming [19, 21, 23, 26, 29, 30]. These elements include universal or system-wide change programs, active involvement of family and community, interactive programming activities, teacher training and support, skills training, relationship-building elements, adequate delivery lengths, and cultural relevancy [19, 21, 23, 26, 29, 30].  Figure 1 presents a graphic summary of the research on the effectiveness of prevention programs and their findings regarding key elements. The following sections constitute a brief description of how the Choices program incorporates these elements.

#### 1.1.1. Universal or System-Wide Change Programs

Including the entire student body, family, school, and community produces larger and longer lasting effects on the high-risk behaviors of students who participate in such programs [25, 26, 30]. The Choices program targets audiences that correspond to all of the IOM's target audience categories: universal, selective, and indicated [28]. Additionally, the program features a system-wide approach offering programming to students, faculty, and parents. During the first Choices implementation year, student action groups, parent action groups, and faculty action groups are formed to decide which risky behavior topics are most culturally relevant and to assist in program implementation throughout the year.

#### 1.1.2. Active Involvement of Family and Community

Prevention outcome research indicates that change is more likely to occur when students practice new skills in dynamic interactions throughout the entire school system, including peers, parents, and community [19, 26, 27, 32–34]. Therefore, Choices includes faculty, parents, and students in all programming. Each group is surveyed prior to program commencement and at the end of each school year to assess opinion and prevalence rates regarding 15 different risky behaviors. Choices programming is offered to all three groups throughout the year including events that bring these groups together.

#### 1.1.3. Interactive Programming Activities

In the extant literature, interactive teaching techniques emerged as an essential element, with interactive programs showing a 21% reduction in risky behaviors prevalence rates as opposed to 4% for noninteractive programs [28]. The Choices program includes interactive programming by utilizing human-to-human and computer-to-human prevention program activities. The faculty, parent, and student action groups cocreate speaking events, plays, lunch-room events, contests, and bulletin board and poster campaigns implemented on a weekly or monthly basis. Student and adult speakers are utilized to share their own experiences, knowledge, or life skills regarding engaging or not engaging in a risky behavior. Interactive Choices program training is offered to home-room, advisory, and health class faculty members who assist in teaching Choices topics.

#### 1.1.4. Teacher Training and Support

After 2 years of Choices programming, a random sample of faculty members chosen to facilitate Choices activities was interviewed in order to understand their lived experiences of participating in program implementation [35]. The investigators found that some teachers admitted that they did not follow exact protocol when facilitating the program activities due to discomfort with topic or narrow teaching options. Thus, a variety of new implementation techniques was created depending upon a teacher's preference that included lecture, role-play/video, or discussion technique. In addition, faculty training on each risky behavior is offered. Faculty handouts including definitions, prevalence rates, and helpful resources regarding each risky behavior are delivered monthly in either paper format, digital format (i.e., via email), or both. Teacher training is offered at faculty in-services and advisory meetings.

#### 1.1.5. Skills Training and Relationship-Building Elements

Choices programming utilizes education and skills training for all prevention programming and activities. Educational components include refusal skills, improving executive functioning skills, and healthy coping skills. Life skills, social and emotional, and positive behavior skills training are integrated and tailored to fit the school's culture. Relationship building is emphasized through the creation of Choices faculty, parent, and student collaborative work groups.

#### 1.1.6. Cultural Relevancy and Adequate Delivery Lengths

Guided by Bronfenbrenner's ecological systems theory, the program curriculum is based on an integrated theoretical perspective that considers behavior as the result of complex interactions among person-, situation-, and environment-level variables within a community [36]. The Choices counselor utilizes a systemic view of the school to collect and to utilize information from all proximal student systems (i.e., individual, peer, family, and community). Cultural relevancy is achieved by tailoring programming to the specific risks of the target population gleaned from quantitative and qualitative surveys as well as via professional integration into the school's system throughout the year.

## 2. Method

This study represented a quasi-experimental, longitudinal design conducted in a medium-sized private high school in a large metropolitan city in the Southwest United States, with national survey data serving as the nonrandomized control group for 2 of the 4 years. The national data include public and private school students and, thus, served as an appropriate comparison group for this study's local private school population because it allowed the researchers to situate the findings within a national context, thereby enhancing the external validity of the findings. The following research questions were addressed: (a) what is the difference between the prevalence rates of risky behaviors for the youth who participate in the program and the national rates of the same year? and (b) what is the difference between pretest data and each subsequent year's data of the youth who participate in the program? Permission to conduct the research was obtained through a local university institutional review board, the school, and the local agency providing the program.

### 2.1. Prevention Activities

During each year of the program, 4 to 6 universal prevention presentations were offered to the entire student body, parents, and faculty/administration depending upon allotted time determined by the school. The presentation topics were chosen based on the risky behaviors having the highest prevalence rates in the previous year's survey. After each school-wide presentation, all students engaged in a topic discussion or activity during their advisory or home-room class. In addition, prevention activities, groups, and events were held each month. Once again, these activity topics were chosen with regard to survey results or current needs determined by school administration and offered to selective groups of students, faculty members, and parents. Indicated students were seen each day, on an as-needed basis, by the prevention program counselor. Also, interactive prevention program presentations regarding each culturally relevant topic were offered each semester during health classes.

### 2.2. Sample

Student risky behaviors were measured via a convenience sample of all students available to respond to the survey given once per year. Participants included all students enrolled in the school from May 2009 to May 2013. Students and parents were given consent forms as a procedure of school enrollment. In the 2008-2009 school year, the Pretest Year, 93.1% of the student body responded to the survey. In the 2009-2010 school year, 86% of the student body responded to the survey, and 70.6% of the student body responded to the survey in the 2010-2011 school year. In the 2011-2012 school year, 84.6% of the student body responded to the survey, and 83.9% of the student body responded to the survey in the 2012-2013 school year.  Table 1 reports descriptive information concerning the student body, including respondent gender, grade, ethnicity, and total separated by survey year.

### 2.3. Measures

The Youth Risk Behavior Survey (YRBS) was utilized to collect a pretest measure in 2009 and 4 years of students' behavioral data after the implementation of the prevention program between 2010 and 2013. Developed by the Centers for Disease Control and Prevention (CDC), the YRBS serves as a national source of information about risky behaviors among adolescents in Grades 9 to 12 and has been given to randomly selected public and private schools in the United States every 2 years since 1991 [1]. No special permission is required to use or to modify the YRBS [37]. The survey consists of 86 multiple-choice or yes/no response format items. National YRBS data are representative of all public and private school students in Grades 9 to 12 in the 50 states and the District of Columbia. Thus, the YRBS is an appropriate comparison group for this study due to the lack of private school-only national databank. YRBS data are weighted to adjust for school and student nonresponse and to make the data representative of the population of students from which the sample was drawn. Generally, these adjustments are made by applying a weight based on student sex, grade, and race/ethnicity [1]. The CDC has conducted score reliability and score validity testing of the 1992 and 2000 versions of the YRBS questionnaires. Regarding test-retest reliability of the YRBS scores, researchers computed kappa statistics for items measuring health risk behaviors and compared group prevalence estimates at two testing occasions [1]. Approximately three-fourths of the survey items were rated as having a substantial or higher score reliability (kappa > 61%) and no statistically significant differences between the prevalence estimates for the first and second times of administration [1]. Researchers at the CDC (2013) indicated that the YRBS provides several types of score validity which reinforces the score reliability of the survey comprising (a) constructs of adolescent behaviors, (b) internal consistency of responses, (c) external validity of causal relationships that are generalizable across populations, and (d) face validity that establishes intent and purpose of the study. Overall, high levels of reliability and validity of the YRBS scores illustrated that prevalence rates within group changes measure 2% across two administrations of the YRBS [1].

However, to address the specific goals of this study, the program developer added items pertaining to additional risky behaviors not included in the YRBS (e.g., pornography, gambling, self-injury, video game use, and date rape). The additional topic items were constructed in an identical format to the existing YRBS item format. In addition, certain original items in the YRBS that did not apply to the present sample were excluded from the survey (e.g., seatbelt or helmet safety questions, dietary questions, and physical activity questions). The YRBS questions regarding bullying, date rape, and dating violence query both whether the student has perpetrated and whether he/she has been the victim of the act. For the purpose of this study, only questions wherein the student endorsed being a victim of the act were utilized for analysis.

### 2.4. Analyses

The quantitative analysis of data included examining the differences between the student data and the national CDC sample data trends as well as differences between the pretest survey data and each subsequent year of the student surveys. The independent variable was time (i.e., before measure versus 1–4 years after measure). The dependent variables, namely, (a) selected student risky behaviors of the local students (i.e., one dependent variable for each risky behavior for the univariate analyses) and (b) the type of students (i.e., the local students versus the national students), were measured as categorical variables. Therefore, a chi-square test was employed to compare pretest data (2009) and study Years 1–4 data (2010–2013) for 20 risky behaviors variables (representing the 15 different risky behaviors), using the Bonferroni adjustment (i.e., .05/20 = .0025) to ensure that the total experiment-wise error rate did not exceed 5% [38].  Table 2 lists the 15 risky behaviors and their 20 corresponding variables. A Bonferroni-adjusted alpha level (*α*) of .0025 was used as the criterion for testing the hypotheses. Frequency scales for each variable yielded categorical data with each participant contributing data to one cell. With regard to the survey data, all variables with out-of-range values and cases with more than one logical inconsistency were removed (e.g., respondents who reported that they had used alcohol within the previous 30 days but never in their lifetimes), which decreased the sample sizes by 1% for Pretest Year (2009), 1.4% for Year 1 (2010), 2% for Year 2 (2011), 2.6% for Year 3 (2012), and 2.2% for Year 4 (2013). Finally, a logistic regression analysis was conducted on each high-risk behavior variable to control for gender and grade. It should be noted that because grade variable was not binary—containing four (i.e., *k*) levels, namely, freshman, sophomore, junior, and senior—it was inappropriate to include the grade variable in the logistic regression model as either an interval-scale or ratio-scale variable. Thus, as recommended by many statisticians [39], three (i.e., *k* − 1) dummy variables (i.e., design variables) were created for each logistic regression model, using freshman as the default (i.e., base) group for each dummy variable.

## 3. Results

### 3.1. National versus Local

The national YRBS survey did not assess some of the risky behaviors that the local modified YRBS did (e.g., gambling, pornography, self-injury, date rape, and video game use) yielding 10 risky behaviors (13 variables) in common. To address Research Question 1, a series (i.e., *n* = 13) of 2 (time) × 2 (risk behavior) chi-square analyses revealed statistically significant differences in the local survey data when compared to national survey data for seven of the 13 variables of interest.  Table 3 lists the percentage of risky behaviors including the amount of change for each variable in Pretest Year (2009), Year 2 (2011), and Year 4 (2013) compared to national rates of the same year.

In Pretest Year 2009, three variables displayed statistically significant differences above national rates: drinking at least one drink within the past 30 days (current drinking), drinking five or more alcoholic drinks on a single occasion within the last 30 days (binge drinking), and drinking and driving. In the same year, four variables displayed statistically significant differences lower than national rates: fasting as diet method within the past 30 days, being bullied within the past 12 months, suicide attempts, and sex/oral sex. In 2011, one variable yielded statistically significant differences above national rates: binge drinking. In the same year, six variables yielded statistically significant differences below national rates: lifetime marijuana use, marijuana use within the last 30 days, cigarettes within the last 30 days, fasting as diet method within the past 30 days, suicide attempts, and sex/oral sex. In 2013, two variables yielded statistically significant differences above national rates: current drinking and binge drinking. In the same year, seven variables yielded statistically significant differences below national rates: lifetime marijuana use, marijuana use within the last 30 days, lifetime cocaine use, fasting as diet method within the past 30 days, suicide attempts, sex/oral sex, and dating violence. The effect sizes for all variables in all years were small.

### 3.2. Pretest Data versus Subsequent Years

When compared to the Pretest Year, nine out of 20 risky variables representing seven risky behaviors displayed statistically significant decreases for each of the 4 years of the study, eight of which were substance-use behaviors. Lifetime alcohol use, current drinking, binge drinking, and drinking and driving all decreased statistically significantly each subsequent year of the study when compared to the Pretest Year. The same result was achieved for lifetime marijuana use, current marijuana use, lifetime cocaine use, and current cigarette use. These results indicate that the prevention program likely had a positive effect on reducing substance use.

However, non-substance-use behaviors yielded variable results, an indicator that the program likely had little effect on these behaviors. Regarding eating disordered behaviors, more students reported engaging in fasting as a diet method each year of the program when compared to the Pretest Year 2009. The same was true for suicide attempts, although no increases were statistically significant for either behavior. The experience of being bullied was statistically significantly higher in each subsequent year of the study when compared to the Pretest Year, doubling by 2013. Regarding being a victim of dating violence, students reported a statistically significant increase in Year 1, followed by two decreases in Year 2 and Year 3, ending in a statistically significant drop in Year 4 when compared to the pretest scores. Video game use decreased statistically significantly each year of the program. However, the number of students who engaged in 5 or more hours of video game use per day statistically significantly increased in Years 1, 2, and 4. Though not statistically significant, students reported decreasing rates of gambling during each year of the program. However, of those who gambled, the rates at which students reported attempting to win back their money increased in Year 1, decreased statistically significantly in Year 2, and increased statistically significantly in Year 3 and Year 4. Another conflicting result was the reported use of pornography, which statistically nonsignificantly decreased in Year 1, statistically significantly decreased in Year 2, and statistically significantly increased in Year 4. The incidence of reported date rape appeared to remain stable except for a statistically significant increase in Year 2.  Table 4 lists the percentage of risky behaviors per each year of the study as compared to the Pretest Year.

### 3.3. Predictors of Change

Table 5 reports the results of the logistic regression analysis of gender, grade, and subsequent years of study for each high-risk variable. Regarding lifetime and current alcohol use, grade and years in the study statistically significantly predicted prevalence rates. Based on odds ratios, seniors were 6.28 times more likely to drink in their lifetimes (*z* = 162.25, *p* < .001) and 8.49 times more likely to currently use alcohol (*z* = 290.71, *p* < .001) than were freshmen. Overall, students' likelihood of lifetime alcohol (*z* = 12.43, *p* < .001) and current alcohol use (*z* = 10.44, *p* < .001) decreased from 2009 to 2013 irrespective of gender and grade. With regard to binge drinking and drinking and driving, it appears that gender and grade had predictive value, as did years in the study. Girls tended to binge-drink more often than did boys and seniors were much more likely to drink and drive (*z* = 133.54, *p* < .001). Over the 4-year span of the study, all students were less likely to engage in either binge drinking (pretest to 2010, *z* = 13.10, *p* < .001 to pretest to 2013, *z* = 7.09, *p* < .001) or drinking and driving (pretest to 2010, *z* = 23.56, *p* < .001 to pretest to 2013, *z* = 12.03, *p* < .001). For binge drinking, the proportion of variance explained, as measured by* Nagelkerke's  R* ^2^, was moderate at 13.3%. The overall prediction success rate was 70%, which comfortably exceeded chance. For drinking and driving, the proportion of variance explained, as measured by* Nagelkerke's  R* ^2^, was moderate at 16.6%. The overall prediction success rate was 85.7%, which comfortably exceeded chance.

Concerning other drugs, grade, gender, and years in the study contributed to the change in lifetime and current marijuana as well as cigarette use. Girls were more likely to report lifetime and current marijuana use (*z* = 4.56, *p* < .05 and *z* = 10.18, *p* < .001, resp.). Contrastingly, boys were more likely to currently use cigarettes (*z* = 16.87, *p* < .001). Seniors were more likely to use marijuana in their lifetimes (*z* = 221.50, *p* < .001) and currently use marijuana (*z* = 142.67, *p* < .001) as well as cigarettes (*z* = 87.53, *p* < .001). For cocaine use, grade and years in study contributed to the variance in prevalence rates. Once again, seniors were more likely to use cocaine (*z* = 32.18, *p* < .001; *z* = 18.08, *p* < .001). There was a significant downward trend in prevalence rates for each of these high-risk behaviors over the span of the study. The odds of lifetime marijuana use changed from 2.25 to 1.39 (*z* = 20.07, *p* < .001; *z* = 18.08, *p* < .001); current marijuana use from 3.18 to 1.41 (*z* = 30.13, *p* < .001; *z* = 14.28, *p* < .001); current cigarette use from 2.32 to 1.49 (*z* = 14.21, *p* < .001; *z* = 16.79, *p* < .001); and cocaine use from 3.59 to 1.44 (*z* = 13.70, *p* < .001; *z* = 6.13, *p* < .001). For lifetime marijuana use, the proportion of variance explained, as measured by Nagelkerke's *R* ^2^, was moderate at 15.2%. The overall prediction success rate was 71.5%, which comfortably exceeded chance. For current marijuana use, the proportion of variance explained, as measured by Nagelkerke's *R* ^2^, was moderate at 15.3%. The overall prediction success rate was 83.7%, which comfortably exceeded chance.

Table 6 reports the results of the logistic regression analysis of gender, grade, and subsequent years of study for each non-substance-use high-risk variable. With regard to non-substance-use high-risk behavior, gender predicted variance in current fasting behaviors, suicide attempts, gambling behaviors, pornography use, self-injury, sex/oral sex, and dating violence. Girls were more likely to endorse the use of fasting to reduce weight (*z* = 29.03, *p* < .001), suicide attempts (*z* = 11.46, *p* < .001), and self-injury (*z* = 16.47, *p* < .001). Contrastingly, boys were more likely to report gambling (*z* = 150.89, *p* < .001), pornography use (*z* = 447.47, *p* < .001), video game use (*z* = 447.30, *p* < .001), and video game use for 5 hours or more per day (*z* = 20.03, *p* < .001). Grade appeared to have a small, varied influence on some behaviors such as current fasting, bullying, suicide, pornography use, self-injury, and dating violence. Juniors and seniors were more likely to experience being bullied (*z* = 14.31, *p* < .05 and *z* = 13.92, *p* < .001, resp.) and to use pornography (*z* = 8.08, *p* < .001 and *z* = 21.58, *p* < .001, resp.). Sophomores appeared more likely to report playing video games for 5 hours or more (*z* = 9.12, *p* < .001). Juniors were more likely to endorse fasting as a weight loss method (*z* = 5.00, *p* < .05). Lastly, seniors were more likely to report attempting suicide within the past year (*z* = 6.38, *p* < .01).

Years in the study contributed to significant variance in bullying, pornography use, and video game use. Even though the percentage of students who reported being bullied was higher for each year of the study when compared to the Pretest Year, the odds of being bullied decreased each year from 2.97 to 1.99 to 1.75 to 1.46 (*z* = 17.30, *p* < .001; *z* = 14.90, *p* < .001; *z* = 16.79, *p* < .001; and *z* = 11.79, *p* < .001, resp.). Odds of pornography use went from 1.55 in the first study year (*z* = 4.31, *p* < .05) to 1.30 in the third and fourth study years (*z* = 5.47, *p* < .05 and *z* = 8.30, *p* < .001, resp.). For pornography use, the proportion of variance explained, as measured by Nagelkerke's *R* ^2^, was large at 33.1%. The overall prediction success rate was 77.5%, which comfortably exceeded chance. Finally, throughout the 4-year study, the odds of video game use decreased and then increased from 6.57 to 1.82 to 2.65 to 2.14 (*z* = 91.37, *p* < .001; *z* = 22.48, *p* < .001; *z* = 88.30, *p* < .001; and *z* = 82.73, *p* < .001, resp.).

## 4. Discussion

This study set out to explore the effects of Choices, a comprehensive prevention program involving all three IOM's categories and targeting multiple risky behaviors that adolescents struggle with today. By comparing the local to the national population that served as a quasi-experimental control group, the data indicated that, prior to the introduction of the prevention program, the local students reported relatively more of a problem with alcohol use behaviors, but relatively less of an issue with eating disorders, bullying, suicide, and sex, and comparable problems with marijuana and other drugs. These results might be a reflection of socioeconomic standing (SES). Problematic alcohol consumption has been associated with higher SES [40] while poor mental health has been associated with lower SES [41]. There is evidence that religious private schooling reduces involvement in most risky behaviors such as sexual activity, criminal arrests, and use of hard drugs, but not drinking, smoking, and marijuana use [42].

After 2 years of prevention programming, the local students reported significant decreases in problem drinking behaviors, marijuana, and tobacco, eating disordered behavior, suicide, and sex problems. By the fourth year of programming, the local students continued to exhibit further decreases in alcohol, drug, and other risky behaviors. Although drinking behaviors remained a major problem, the prevalence of alcohol use did not reach pretest difference levels and drinking and driving behavior maintained large decreases comparable to national data. The local population also displayed sizable significant decreases in marijuana use compared to the national students.

Internally, when comparing the local population each year of the study, 12 risky behavior variables decreased—nine statistically significantly—from pretest to Year 4. Eight of these variables were alcohol and drug related to a significant portion of the variance attributable to years in the study. Thus, Choices likely had a positive effect on reducing substance use. This is an important finding in light of the research which indicates that adolescents who start using drugs and alcohol before the age of 18 have a one-in-four chance of becoming addicted, compared with a one-in-25 chance for those who began at the age of 21 or later [29]. Moreover, this result is consistent with a recent systematic review of multiple health risk behaviors interventions which indicated that the highest reductions in risky behavior were seen in various forms of substance use in these programs [23].

Unfortunately, the results of this study revealed inconsistent outcomes for the other non-substance-use risky behaviors targeted by the Choices program. The local population in this study reported lower incidences of non-substance-use risky behaviors than did the national population. However, during the study period, four of these variables increased, one statistically significantly. Five others displayed increases and decreases from pretest to Year 4. Thus, it is likely that the program had little effect on non-substance-use risky behaviors.

Perhaps the decreases in substance-use rates suggest that the programming dosage levels for substance-use behaviors were adequate and might have assisted in achieving the positive outcomes. In fact, the amount of substance-use programming was greater due to the school's programming needs as indicated in the student surveys. A recent review of multiple health risk behavior prevention interventions suggests that programs that target multiple substance-use behaviors also can be effective for reducing other risky behaviors [23]. However, one possible implication is that dosage levels for non-substance-use behaviors should be increased at least to that of the substance-use behaviors. Another possible assumption is that the effects of the underlying mechanisms of the program such as skills building and social competency might take time to manifest as participants learn and employ these skills. Yet a third possible conclusion is that the underlying causes of the non-substance-use risky behaviors are different from those of substance-related risky behaviors. Research affirms conventional wisdom that diverse risky behaviors are correlated but not strongly and approximately two-thirds of the variance in risky behaviors is attributable to unique rather than the same causes [43]. An examination of the cooccurrence and predictors of risky behavior in late adolescence identified a low-risk group, a high-risk group, and two moderate-risk groups consisting of different combinations of risky behaviors [21]. This evidence highlights the necessity for examining the complexity of relationships among multiple risky behaviors to help understand why interventions that have an effect on one might not affect others.

The preliminary results of this study demonstrate the effectiveness of a comprehensive, multiple risky behaviors prevention program for substance-use behaviors and the potential for success on non-substance-use behaviors. However, only a top-level statistical analysis was completed for this study. Future examination of such a program could be improved by utilizing a control group, comparing each year's survey data to the previous year in addition to the Pretest Year, comparing individual student survey responses over time, utilizing a cross-sectional study design, and increasing the frequency and consistency of non-substance-use programming in addition to examining the potential mediating effects such as random drug testing initiatives, dosage levels, student maturation, and peer norms. Also, future studies should adjust for socioeconomic status (SES), previous exposure to prevention programming, ethnicity, family variables, and attrition of students. Confounding variables such as the incoming freshman and outgoing seniors should be examined for year-to-year population differences as well as those students who drop out of school and why. An additional limitation of this study includes no reliability or validity ratings for the added survey questions (e.g., pornography; gambling; self-injury; video game use; date rape) included in the modified survey instrument. This study possesses limited generalizability due to the private school student body.

## 5. Conclusion

To conclude, in part due to the modern technological advances of our day, students are exposed earlier in life to a broad range of potentially addictive behaviors, including eating disorders, gambling, Internet, love, sex, exercise, work, and shopping [4]. The choices that students make to engage or not to engage in such behaviors occur within and are influenced by the many levels of their social systems. Thus, prevention programs must keep up with the trends occurring in modern student environments and integrate programming into multiple levels of students' social systems. Of course, school administrators could choose to purchase separate prevention programs specifically to target each non-substance-use behavior; however, this is not recommended for several reasons. Previous research indicates that an integrated model of prevention is preferred in order to (a) address adequately the underlying mechanisms contributing to the behavior, (b) maximize intervention exposure, (c) create an additive or multiplicative effect by blending evidence-based strategies, (d) reduce system overload and maximize sustainability, and (e) maintain implementation quality [32]. However, an important implication drawn from this study is that when proponents of school-based prevention encourage schools to develop such comprehensive models, they take into consideration the school's ability to allow ample time for adequate coverage and saturation of all relevant risky behavior topics. For prevention specialists and researchers, this study suggests that further examination of the underlying causes of non-substance-use risky behaviors, as well as prevention program dosage levels, to help understand why interventions that have an effect on one behavior might not affect others, is warranted.

## Figures and Tables

**Table 1:** Demographics of student body and survey respondents by gender, grade, ethnicity, and total by year of study programs.

	2009				2010				2011				2012				2013			
	Student body		Survey respondent		Student body		Survey respondent		Student body		Survey respondent		Student body		Survey respondent		Student body		Survey respondent	
	*N* = 659		*N* = 614		*N* = 661		*N* = 569		*N* = 664		*N* = 469		*N* = 679		*N* = 575		*N* = 673		*N* = 565	
	*n*	%	*n*	%	*n*	%	*n*	%	*n*	%	*n*	%	*n*	%	*n*	%	*n*	%	*n*	%
Gender																				
Male	316	48	306	49.8	308	46.6	252	44.3	292	44	208	44.3	309	45.5	266	46.3	331	49.2	275	50.0
Female	343	52	308	50.2	353	53.4	317	55.7	372	56	261	55.7	370	54.5	309	53.7	342	50.8	290	50.0
Grade																				
9th	167	25.4	154	25.1	171	25.9	154	27.1	163	24.5	144	30.7	166	24.4	158	27.5	164	24.4	154	30.0
10th	165	25.0	154	25.1	168	25.4	156	27.4	176	26.5	110	23.5	169	24.9	156	27.1	166	24.7	143	30.0
11th	165	25.0	158	25.7	160	24.2	144	25.3	169	25.5	125	26.7	173	25.5	123	21.4	172	25.6	159	30.0
12th	162	24.6	148	24.1	162	24.5	115	20.2	156	23.5	90	19.2	171	25.2	138	24.0	171	25.4	109	20.0
Ethnicity																				
African American	38	5.8	—	—	41	6.2	—	—	50	7.5	—	—	52	7.7	—	—	55	8.2	—	—
Asian	23	3.5	—	—	22	3.3	—	—	25	3.8	—	—	21	3.1	—	—	18	2.7	—	—
Latino/Hispanic	48	7.3	—	—	43	6.5	—	—	43	6.5	—	—	39	5.7	—	—	48	7.1	—	—
Middle Eastern	13	2	—	—	12	1.8	—	—	11	1.7	—	—	6	0.9	—	—	9	1.3	—	—
Multiracial	0	0	—	—	4	0.6	—	—	2	0.3	—	—	12	1.8	—	—	11	1.6	—	—
International	0	0	—	—	7	1.1	—	—	7	1.1	—	—	12	1.8	—	—	10	1.5	—	—
White	534	81	—	—	532	80.5	—	—	526	79.2	—	—	536	78.9	—	—	521	77.4	—	—
Other	3	0.5	—	—	0	0	—	—	0	0	—	—	1	0.1	—	—	1	0.1	—	—

*Note.* Dash mark indicates data not reported.

**Table 2:** High-risk behaviors and corresponding variables.

High-risk behavior	Corresponding variables
(1) Alcohol use	Lifetime alcohol
	Drinking 1 or more times in the past 30 days
	Drinking 5 or more times in the past 30 days

(2) Drinking and driving	Drinking and driving in the past year

(3) Marijuana use	Lifetime marijuana use
	Marijuana use in the past 30 days

(4) Heavy drug use	Lifetime cocaine use

(5) Cigarette use	Cigarette use in the past 30 days

(6) Eating disordered behavior	Fasting in the past 30 days

(7) Bullying	Bullied in the past year

(8) Suicide	Suicide attempts in the past year

(9) Sex/oral sex	Sex/oral sex in the past year

(10) Dating violence	Dating violence in the past year

(11) Date rape	Date rape in the past year

(12) Gambling	Gambling in the past year
	Tried to win back money

(13) Pornography use	Pornography in the past 30 days

(14) Self-injury	Self-injury in the past year

(15) Video game overuse	Video game use
	Video game use for 5 or more hours

**Table 3:** Local versus national risk behavior as a percentage of the sample: Pretest year (2009), Year 2 (2011), and Year 4 (2013).

Risk behavior	Pretest year (2009)			Year 2 (2011)			Year 4 (2013)		
	Local (*n* = 614)	National (*n* = 16,410)	Difference	Local (*n* = 469)	National (*n* = 15,425)	Difference	Local (*n* = 565)	National (*n* = 13,583)	Difference
	(%)	(%)	(%)	(%)	(%)	(%)	(%)	(%)	(%)
Lifetime alcohol use	76.2	73.9	2.3	71.0	68.4	2.6	67.0	67.7	−0.7
Drinking in the last 30 days	54.2	42.0	12.2^*∗*^	43.7	39.0	4.7	43.4	35.6	7.8^*∗*^
Drinking 5 or more times in the last 30 days	38.3	24.0	14.3^*∗*^	27.5	21.9	5.6^*∗*^	27.8	20.1	7.7^*∗*^
Drinking and driving in the last year	21.0	9.9	11.1^*∗*^	10.7	8.3	2.4	10.3	9.7	0.6
Lifetime marijuana use	38.3	38.3	0.0	27.7	39.9	−12.2^*∗*^	28.7	43.7	−15.0^*∗*^
Marijuana in the last 30 days	24.6	20.9	3.7	16.0	23.6	−7.6^*∗*^	9.9	25.3	−15.4^*∗*^
Lifetime cocaine use	7.7	7.3	0.4	5.3	7.2	−1.9	2.5	5.6	−3.1^*∗*^
Cigarettes in the last 30 days	19.4	18.9	0.5	11.7	17.2	−5.5^*∗*^	14.9	14.5	0.4
Fasting in the last 30 days	5.4	11.1	−5.7^*∗*^	7.0	12.7	−5.7^*∗*^	6.9	13.3	−6.4^*∗*^
Bullied last year	9.9	19.0	−9.1^*∗*^	17.5	18.0	−0.5	20.0	18.6	1.4
Suicide attempts in the last year	1.3	7.2	−5.9^*∗*^	3.4	8.7	−5.3^*∗*^	4.3	8.5	−4.2^*∗*^
Sex/oral sex in the last year	38.1	50.4	−12.3^*∗*^	31.1	50.5	−19.4^*∗*^	32.4	49.7	−17.3^*∗*^
Dating violence	8.5	11.1	−2.6	6.6	10.5	−3.9	4.6	10.7	−6.1^*∗*^

*Note.* The national survey is administered every two years, thereby limiting the comparison between local and national data to every two years.

^*∗*^ *p* < .005.

**Table 4:** Risk behavior per year of study as a percentage of the sample—all years.

Risk behavior	2009 (*n* = 614)	2010 (*n* = 569)	2011 (*n* = 469)	2012 (*n* = 575)	2013 (*n* = 565)
	(%)	(%)	(%)	(%)	(%)
Lifetime alcohol use	76.2	68.0^d^	68.4^d^	68.0^d^	67.1^d^
Drinking in the last 30 days	54.2	45.3^d^	43.7^d^	43.7^d^	43.5^d^
Drinking 5 or more times in the last 30 days	38.3	33.2^d^	27.5^d^	29.4^d^	28.0^d^
Drinking and driving in the last year	21.0	15.8^d^	10.7^d^	12.5^d^	10.4^d^
Lifetime marijuana use	38.3	28.1^d^	27.7^d^	26.1^d^	28.8^d^
Marijuana in the last 30 days	24.6	16.5^d^	16.0^d^	13.4^d^	10.1^d^
Lifetime cocaine use	7.7	5.4^d^	5.3^d^	2.1^d^	2.7^d^
Cigarettes in the last 30 days	19.4	11.4^d^	11.7^d^	12.2^d^	15.0^d^
Fasting in the last 30 days	5.4	7.7	7.0	6.6	7.1
Bullied last year	9.9	16.0^u^	17.5^u^	15.3^u^	20.2^u^
Suicide attempts in the last year	1.3	3.9	3.4	3.3	4.4
Gambling in the last year	22.6	19.5	17.5	18.6	20.2
Winning back money	8.1	9.0	2.1^d^	11.8^u^	14.7^u^
Pornography in the last 30 days	28.3	21.8	17.5^d^	25.0	29.7^u^
Self-injury in the last year	12.4	10.4	8.3	9.9	9.6
Sex/oral sex in the last year	38.1	32.3	31.1	32.0	32.6
Dating violence	8.5	16.5^u^	6.6	5.2	4.8^d^
Date rape	0.7	0.7	2.3^u^	0.9	1.1
Video game use	49.8	38.8^d^	10.7^d^	42.3^d^	41.4^d^
Video game use for 5 or more hours	1.5	2.1^u^	2.3^u^	1.2^d^	2.5^u^

*Note.* “d” denotes a decrease and “u” denotes an increase at *p* < .0005 when compared to the Pretest Year.

**Table 5:** Logistic regression analysis of gender, grade, and subsequent study years for substance-use risk behaviors.

	*B*	SE	Wald's test (*z* ratio)	Odds ratio	95% CI
Lifetime alcohol use					
Intercept	−1.09	0.44	6.29^*∗∗*^	0.34	
Gender	−0.15	0.09	3.16	0.86	0.72, 1.02
Sophomore	0.54	0.11	25.34^*∗∗∗*^	1.72	1.39, 2.12
Junior	1.01	0.11	77.85^*∗∗∗*^	2.74	2.19, 3.43
Senior	1.84	0.14	162.25^*∗∗∗*^	6.28	4.73, 8.33
Pretest (2009) to 2010	0.68	0.19	12.43^*∗∗∗*^	1.98	1.35, 2.90
Pretest (2009) to 2011	0.47	0.13	12.72^*∗∗∗*^	1.61	1.24, 2.08
Pretest (2009) to 2012	0.34	0.10	10.95^*∗∗∗*^	1.40	1.15, 1.71
Pretest (2009) to 2013	0.27	0.08	10.44^*∗∗∗*^	1.30	1.11, 1.53
Drinking in the last 30 days					
Intercept	−2.41	0.40	36.45^*∗∗∗*^	0.09	
Gender	−0.08	0.08	1.01	0.92	0.79, 1.08
Sophomore	0.71	0.11	38.57^*∗∗∗*^	2.03	1.62, 2.53
Junior	1.22	0.11	115.79^*∗∗∗*^	3.38	2.70, 4.22
Senior	2.14	0.13	290.71^*∗∗∗*^	8.49	6.36, 10.85
Pretest (2009) to 2010	0.70	0.17	15.87^*∗∗∗*^	2.01	1.42, 2.83
Pretest (2009) to 2011	0.45	0.12	14.15^*∗∗∗*^	1.57	1.24, 1.99
Pretest (2009) to 2012	0.32	0.09	12.00^*∗∗∗*^	1.38	1.15, 1.65
Pretest (2009) to 2013	0.26	0.07	12.39^*∗∗∗*^	1.30	1.12, 1.50
Drinking 5 or more times in the last 30 days					
Intercept	−3.35	0.42	65.01^*∗∗∗*^	0.03	
Gender	0.23	0.09	7.08^*∗∗*^	1.26	1.06, 1.49
Sophomore	0.85	0.13	39.95^*∗∗∗*^	2.34	1.80, 3.05
Junior	1.31	0.13	99.47^*∗∗∗*^	3.69	2.86, 4.77
Senior	1.89	0.13	201.67^*∗∗∗*^	6.64	5.11, 8.63
Pretest (2009) to 2010	0.65	0.18	13.10^*∗∗∗*^	1.91	1.35, 2.72
Pretest (2009) to 2011	0.34	0.12	7.922^*∗∗∗*^	1.41	1.11, 1.79
Pretest (2009) to 2012	0.27	0.09	8.012^*∗∗∗*^	1.31	1.09, 1.58
Pretest (2009) to 2013	0.20	0.08	7.09^*∗∗*^	1.23	1.06, 1.43
Drinking and driving in the last year					
Intercept	−6.02	0.55	119.13^*∗∗∗*^	0.00	
Gender	0.40	0.11	12.43^*∗∗∗*^	1.50	1.20, 1.87
Sophomore	1.20	0.24	25.37^*∗∗∗*^	3.33	2.09, 5.32
Junior	1.75	0.23	58.42^*∗∗∗*^	5.73	3.66, 8.97
Senior	2.57	0.22	133.54^*∗∗∗*^	13.11	8.48, 20.29
Pretest (2009) to 2010	1.09	0.23	23.56^*∗∗∗*^	2.99	1.92, 4.65
Pretest (2009) to 2011	0.58	0.15	14.418^*∗∗∗*^	1.78	1.322, 2.40
Pretest (2009) to 2012	0.46	0.12	14.93^*∗∗∗*^	1.58	1.25, 2.00
Pretest (2009) to 2013	0.33	0.10	12.03^*∗∗∗*^	1.40	1.16, 1.69
Lifetime marijuana use					
Intercept	−4.00	0.43	87.96	0.02	
Gender	0.19	0.09	4.56^*∗*^	1.21	1.02, 1.43
Sophomore	0.91	0.14	40.63^*∗∗∗*^	2.48	1.87, 3.27
Junior	1.46	0.14	112.97^*∗∗∗*^	4.30	3.29, 5.63
Senior	2.08	0.14	221.50^*∗∗∗*^	7.97	6.07, 10.48
Pretest (2009) to 2010	0.81	0.18	20.07^*∗∗∗*^	2.25	1.58, 3.22
Pretest (2009) to 2011	0.55	0.12	19.12^*∗∗∗*^	1.73	1.35, 2.20
Pretest (2009) to 2012	0.37	0.10	14.75^*∗∗∗*^	1.45	1.20, 1.75
Pretest (2009) to 2013	0.33	0.08	18.08^*∗∗∗*^	1.39	1.20, 1.62
Marijuana in the last 30 days					
Intercept	−5.74	0.51	126.30^*∗∗∗*^	0.00	
Gender	0.35	0.11	10.18^*∗∗∗*^	1.41	1.14, 1.75
Sophomore	1.04	0.20	26.67^*∗∗∗*^	2.83	1.91, 4.21
Junior	1.39	0.19	51.12^*∗∗∗*^	4.03	2.75, 5.91
Senior	2.26	0.19	142.67^*∗∗∗*^	9.57	6.60, 13.89
Pretest (2009) to 2010	1.16	0.21	30.13^*∗∗∗*^	3.18	2.10, 4.80
Pretest (2009) to 2011	0.77	0.14	28.14^*∗∗∗*^	2.16	1.62, 2.87
Pretest (2009) to 2012	0.50	0.11	19.90^*∗∗∗*^	1.65	1.32, 2.05
Pretest (2009) to 2013	0.34	0.09	14.28^*∗∗∗*^	1.41	1.18, 1.68
Lifetime cocaine use					
Intercept	−6.53	0.83	62.27^*∗∗∗*^	0.00	
Gender	0.01	0.18	0.00	1.01	0.071, 1.44
Sophomore	1.11	0.34	10.54^*∗∗∗*^	3.04	1.55, 5.94
Junior	1.00	0.35	8.39^*∗∗∗*^	2.73	1.38, 5.38
Senior	1.84	0.33	32.18^*∗∗∗*^	6.32	3.34, 11.95
Pretest (2009) to 2010	1.28	0.35	13.70^*∗∗∗*^	3.59	1.86, 7.06
Pretest (2009) to 2011	0.86	0.24	13.07^*∗∗∗*^	2.36	1.48, 3.76
Pretest (2009) to 2012	0.38	0.18	4.55^*∗*^	1.46	1.03, 2.06
Pretest (2009) to 2013	0.36	0.15	6.13^*∗∗*^	1.44	1.08, 1.92
Cigarettes in the last 30 days					
Intercept	−5.59	0.54	105.74^*∗∗∗*^	0.00	
Gender	0.46	0.11	16.87^*∗∗∗*^	1.59	1.27, 1.98
Sophomore	1.03	0.20	25.59^*∗∗∗*^	2.81	1.88, 4.18
Junior	1.40	0.20	50.88^*∗∗∗*^	4.06	2.76, 5.97
Senior	1.82	0.19	87.53^*∗∗∗*^	6.17	4.22, 9.04
Pretest (2009) to 2010	0.84	0.22	14.21^*∗∗∗*^	2.32	1.50, 3.60
Pretest (2009) to 2011	0.58	0.15	14.06^*∗∗∗*^	1.79	1.32, 2.42
Pretest (2009) to 2012	0.43	0.12	12.92^*∗∗∗*^	1.54	1.21, 1.95
Pretest (2009) to 2013	0.40	0.10	16.79^*∗∗∗*^	1.49	1.23, 1.81

*Note.* The coefficients for grade contrast with freshman. CI: confidence interval.

^*∗*^ *p* < .05; ^*∗∗*^ *p* < .01; ^*∗∗∗*^ *p* < .001.

**Table 6:** Logistic regression analysis of gender, grade, and subsequent study years for non-substance-use risk behaviors.

	*B*	SE	Wald's test (*z* ratio)	Odds ratio	95% CI
Fasting in the last 30 days					
Intercept	0.30	0.78	0.14	1.35	
Gender	0.91	0.17	29.03^*∗∗∗*^	2.49	1.79, 3.47
Sophomore	−0.22	0.20	1.27	0.80	0.55, 1.18
Junior	0.53	0.24	5.00^*∗*^	1.70	1.07, 2.69
Senior	−0.08	0.21	0.13	0.93	0.61, 1.41
Pretest (2009) to 2010	0.46	0.35	1.71	1.59	0.79, 3.18
Pretest (2009) to 2011	0.34	0.24	1.97	1.40	0.86, 2.24
Pretest (2009) to 2012	0.27	0.18	2.22	1.32	0.92, 1.89
Pretest (2009) to 2013	0.19	0.15	1.67	1.21	0.91, 1.62
Bullied last year					
Intercept	−0.47	0.57	0.66	0.63	
Gender	−0.20	0.11	3.54	0.82	0.67, 1.01
Sophomore	0.02	0.13	0.01	1.02	0.78, 1.32
Junior	0.56	0.15	14.31^*∗∗∗*^	1.75	1.31, 2.35
Senior	0.59	0.16	13.92^*∗∗∗*^	1.81	1.32, 2.46
Pretest (2009) to 2010	1.09	0.26	17.30^*∗∗∗*^	2.97	1.78, 4.95
Pretest (2009) to 2011	0.69	0.18	14.90^*∗∗∗*^	1.99	1.40, 2.83
Pretest (2009) to 2012	0.56	0.14	16.79^*∗∗∗*^	1.75	1.34, 2.28
Pretest (2009) to 2013	0.38	0.11	11.79^*∗∗∗*^	1.46	1.18, 1.81
Suicide attempts last year					
Intercept	−0.80	1.42	0.31	0.45	
Gender	0.76	0.22	11.46^*∗∗∗*^	2.14	1.38, 3.32
Sophomore	0.45	0.32	1.94	1.57	0.83, 2.95
Junior	0.31	0.33	0.88	1.36	0.71, 2.61
Senior	0.79	0.31	6.38^*∗∗*^	2.21	1.20, 4.10
Pretest (2009) to 2010	−1.99	0.67	8.96^*∗∗∗*^	0.14	0.04, 0.50
Pretest (2009) to 2011	−1.36	0.45	9.30^*∗∗∗*^	0.26	0.11, 0.61
Pretest (2009) to 2012	−1.04	0.34	9.44^*∗∗∗*^	0.35	0.18, 0.69
Pretest (2009) to 2013	−0.77	0.27	7.94^*∗∗∗*^	0.46	0.27, 0.79
Gambling last year					
Intercept	−4.10	0.48	73.77^*∗∗∗*^	0.02	
Gender	1.27	0.10	150.89^*∗∗∗*^	3.56	2.91, 4.36
Sophomore	0.05	0.13	0.16	1.05	0.81, 1.37
Junior	0.05	0.13	0.15	1.05	0.81, 1.37
Senior	−0.09	0.14	0.43	0.91	0.69, 1.21
Pretest (2009) to 2010	0.36	0.20	3.12	1.44	0.96, 2.15
Pretest (2009) to 2011	0.19	0.14	1.91	1.21	0.92, 1.60
Pretest (2009) to 2012	0.16	0.11	2.17	1.17	0.95, 1.45
Pretest (2009) to 2013	0.14	0.09	2.61	1.15	0.97, 1.37
Winning money back					
Intercept	6.60	0.81	66.89^*∗∗∗*^	737.67	
Gender	−2.29	0.19	139.17^*∗∗∗*^	0.10	0.07, 0.15
Sophomore	−0.06	0.19	0.08	0.95	0.65, 1.38
Junior	−0.05	0.19	0.08	0.95	0.65, 1.38
Senior	−0.21	0.19	1.18	0.81	0.56, 1.18
Pretest (2009) to 2010	−0.20	0.34	0.35	0.82	0.42, 1.60
Pretest (2009) to 2011	0.39	0.21	3.51	1.47	0.98, 2.21
Pretest (2009) to 2012	−0.17	0.18	0.94	0.84	0.59, 1.19
Pretest (2009) to 2013	−0.19	0.14	1.67	0.83	0.63, 1.10
Pornography in the last 30 days					
Intercept	−6.72	0.52	166.95	0.00	
Gender	2.63	0.12	447.47^*∗∗∗*^	13.86	10.86, 17.68
Sophomore	0.12	0.14	0.66	1.12	0.85, 1.49
Junior	0.40	0.14	8.08^*∗∗∗*^	1.49	1.13, 1.96
Senior	0.66	0.14	21.58^*∗∗∗*^	1.94	1.47, 2.57
Pretest (2009) to 2010	0.44	0.21	4.31^*∗*^	1.55	1.03, 2.36
Pretest (2009) to 2011	0.18	0.14	1.56	1.20	0.90, 1.59
Pretest (2009) to 2012	0.26	0.11	5.47^*∗*^	1.30	1.04, 1.63
Pretest (2009) to 2013	0.26	0.09	8.30^*∗∗∗*^	1.30	1.09, 1.56
Self-injury in the last year					
Intercept	2.75	0.59	21.87^*∗∗∗*^	15.67	
Gender	0.53	0.13	16.47^*∗∗∗*^	1.70	1.31, 2.19
Sophomore	−0.13	0.18	0.50	0.88	0.62, 1.26
Junior	−0.25	0.18	2.00	0.78	0.55, 1.10
Senior	−0.42	0.18	5.31^*∗*^	0.66	0.46, 0.94
Pretest (2009) to 2010	−0.57	0.26	4.91^*∗*^	0.57	0.34, 0.94
Pretest (2009) to 2011	−0.30	0.17	2.96	0.74	0.56, 1.04
Pretest (2009) to 2012	−0.27	0.14	4.00^*∗*^	0.76	0.58, 1.00
Pretest (2009) to 2013	−0.21	0.11	3.75^*∗*^	0.81	0.65, 1.00
Sex/oral sex					
Intercept	3.36	0.42	63.40^*∗∗∗*^	28.85	
Gender	−0.48	0.09	30.73^*∗∗∗*^	0.62	0.53, 0.74
Sophomore	−0.90	0.14	43.94^*∗∗∗*^	0.41	0.31, 0.53
Junior	−1.55	0.13	138.41^*∗∗∗*^	0.21	0.16, 0.28
Senior	−2.12	0.14	244.32^*∗∗∗*^	0.12	0.09, 0.16
Pretest (2009) to 2010	−0.38	0.18	4.30^*∗∗*^	0.69	0.48, 0.98
Pretest (2009) to 2011	−0.24	0.12	3.72^*∗*^	0.79	0.62, 1.00
Pretest (2009) to 2012	−0.18	0.10	3.33	0.84	0.70, 1.01
Pretest (2009) to 2013	−0.15	0.08	3.67	0.86	0.74, 1.00
Dating violence					
Intercept	3.49	0.68	26.60^*∗∗∗*^	32.84	
Gender	−0.78	0.14	29.26^*∗∗∗*^	0.46	0.35, 0.61
Sophomore	−0.29	0.21	1.98	0.75	0.50, 1.12
Junior	−0.15	0.21	0.53	0.86	0.57, 1.30
Senior	−0.75	0.20	14.22^*∗∗∗*^	0.47	0.32, 0.70
Pretest (2009) to 2010	−0.20	0.30	0.43	0.82	0.45, 1.49
Pretest (2009) to 2011	0.21	0.20	1.13	1.24	0.84, 1.83
Pretest (2009) to 2012	0.24	0.15	2.36	1.27	0.94, 1.71
Pretest (2009) to 2013	0.20	0.12	2.67	1.23	0.96, 1.57
Date rape					
Intercept	4.14	2.19	3.56	62.84	
Gender	−0.40	0.37	1.14	0.67	0.32, 1.39
Sophomore	−0.83	0.71	1.35	0.44	0.11, 1.76
Junior	−1.31	0.66	3.92^*∗*^	0.27	0.07, 0.99
Senior	−1.61	0.66	6.04^*∗∗*^	0.20	0.06, 0.72
Pretest (2009) to 2010	1.23	0.96	1.64	3.41	0.52, 22.30
Pretest (2009) to 2011	0.40	0.68	0.35	1.50	0.39, 5.69
Pretest (2009) to 2012	0.56	0.51	1.23	1.76	0.65, 4.78
Pretest (2009) to 2013	0.41	0.41	0.99	1.51	0.67, 3.38
Video game use					
Intercept	−6.76	0.46	216.74^*∗∗∗*^	0.00	
Gender	1.96	0.09	447.30^*∗∗∗*^	7.07	5.90, 8.48
Sophomore	−0.44	0.12	12.78^*∗∗∗*^	0.64	0.51, 0.82
Junior	−0.59	0.13	21.86^*∗∗∗*^	0.56	0.44, 0.71
Senior	−0.55	0.13	18.08^*∗∗∗*^	0.58	0.45, 0.74
Pretest (2009) to 2010	1.88	0.20	91.37^*∗∗∗*^	6.57	4.47, 9.66
Pretest (2009) to 2011	0.60	0.13	22.48^*∗∗∗*^	1.82	1.42, 2.33
Pretest (2009) to 2012	0.97	0.10	88.30^*∗∗∗*^	2.65	2.16, 3.24
Pretest (2009) to 2013	0.76	0.08	82.73^*∗∗∗*^	2.14	1.82, 2.52
Video game use for 5 or more hours					
Intercept	−5.62	1.54	13.25^*∗∗∗*^	0.00	
Gender	1.53	0.34	20.03^*∗∗∗*^	4.63	2.37, 9.05
Sophomore	1.25	0.41	9.12^*∗∗∗*^	3.49	1.55, 7.87
Junior	0.19	0.49	0.16	1.21	0.46, 3.18
Senior	0.65	0.46	2.00	1.92	0.78, 4.76
Pretest (2009) to 2010	−0.67	0.66	1.02	0.51	0.14, 1.88
Pretest (2009) to 2011	−0.39	0.45	0.74	0.68	0.28, 1.65
Pretest (2009) to 2012	−0.49	0.34	2.03	0.61	0.32, 1.20
Pretest (2009) to 2013	−0.24	0.28	0.74	0.79	0.46, 1.36

*Note.* The coefficients for grade contrast with freshman. CI: confidence interval.

^*∗*^ *p* < .05; ^*∗∗*^ *p* < .01; ^*∗∗∗*^ *p* < .001.
